# Impact of immersive virtual reality and transcranial direct current stimulation over the parietal cortex on motor response

**DOI:** 10.3934/Neuroscience.2026019

**Published:** 2026-08-17

**Authors:** Olívia Morgan Lapenta, Rafael de Albuquerque Lima, Gabriela do Amparo Nogueira, Sabrina Baron, Paulo Sérgio Boggio, Marília Lira da Silveira Coelho

**Affiliations:** 1 Psychological Neuroscience Laboratory, Psychology Research Center, School of Psychology, University of Minho - Rua da Universidade, 4710–057 Braga, Portugal; 2 Department of Physical Therapy, Center for Health and Biological Sciences, Mackenzie Presbyterian University, 01241–001, São Paulo, Brazil; 3 Social and Cognitive Neuroscience Laboratory, Mackenzie Presbyterian University - Rua Piauí, 181, 01241–001 São Paulo, Brazil; 4 National Institute of Science and Technology on Social and Affective Neuroscience (INCT-SANI), São Paulo, Brazil

**Keywords:** brain stimulation, virtual reality, motor transfer, multisensory integration, visuomotor tasks, posterior parietal cortex, hand-eye coordination

## Abstract

Immersive virtual reality (IVR) provides interactive and engaging environments to stimulate motor learning and neuroplasticity. Combining IVR with transcranial direct current stimulation (tDCS) may further enhance these effects by modulating activity in targeted brain areas. We investigated the effects of a 5-day IVR game-training (Beat Saber game for PS4) alone or combined with sham or anodal tDCS over the left posterior parietal cortex, a key structure for visuospatial processing and motor coordination. Seventy-four healthy participants were randomly assigned to one of three groups: IVR alone, IVR + anodal tDCS, or IVR + sham tDCS. Participants performed a battery of motor tasks requiring hand–eye coordination (Box and Blocks, Ruler Drop, Trail Making part A, and Plate Tapping tests) before and after training on days 1 and 5 to assess motor performance and transfer effects. Results showed progressive improvements in game performance from day 1 to 4 and significant motor transfer effects across all tasks, regardless of stimulation condition. tDCS did not further enhance motor outcomes, suggesting potential limitations related to stimulation site and/or parameters. Our findings highlight IVR's potential for motor skill development, contributing to the growing body of evidence supporting its use as a versatile and accessible tool for motor learning, research, and rehabilitation. While tDCS did not enhance outcomes in this context, future research should explore alternative protocols to optimize its efficacy in IVR motor training.

## 1. Introduction

Motor learning is a dynamic process that involves the acquisition and consolidation of skills necessary for precise and coordinated movement in performing daily tasks [1]. Enhancing motor performance and optimizing visuomotor response speed are key goals in improving functional efficiency, and these abilities can be developed through structured practice and technological training strategies. Among these, virtual reality (VR) has been widely supported in the literature as an effective technological tool for training motor performance in both healthy and motor/cognitive rehabilitation contexts [2],[3].

VR systems provide an interactive and controlled environment for performing motor tasks, allowing the manipulation of sensory feedback and task difficulty through a user–computer interface. For instance, VR has been used to change visual feedback during reaching movements to see how different errors affect motor learning [4], alter sensory signals to show that a stronger sense of body ownership speeds up task adaptation [5], and adjust task difficulty to test its impact on how well skills are retained and transferred [6]. Within this context, immersive virtual reality (IVR) has been shown to enhance the sense of presence and embodiment by integrating motion tracking and stereoscopic displays, enabling more naturalistic and multisensory interactions within the virtual environment [7].

The use of multimodal feedback during virtual tasks, including visual, auditory, and haptic modalities [8], as well as combined sensorimotor cues [9], promotes continuous sensorimotor adjustments, facilitating spatial adaptation and movement precision. Thus, these diverse feedback mechanisms have been shown to improve precision, reaction time, and movement speed. The enriched, responsive environments not only support visuomotor adaptation but may also enhance engagement and motivation, which are psychological factors associated with optimized motor learning and skill retention [10].

In this study, we used the game Beat Saber within an IVR environment that combines visual, auditory, and sensorimotor stimuli. The task requires visuomotor responses guided by visual cues and synchronized with musical rhythm, promoting immersion within the peripersonal space, a condition that enhances multisensory integration and fine motor adjustment. A recent systematic review highlights that IVR experiences can dynamically modulate and extend peripersonal space boundaries, specifically through virtual tool use and embodiment-related interactions, which in turn enhance spatial awareness and optimize sensorimotor mapping during goal-directed actions [11].

*Beat Saber* involves rapid decision-making, visuospatial attention, and real-time motor control. Players must rapidly anticipate the direction and color of incoming blocks and perform coordinated, rhythmic movements with body rotations and postural adjustments to reach targets at different angles. This combination of cognitive and motor demands makes Beat Saber a valid task to investigate frontoparietal network engagement, and particularly the role of posterior parietal cortex (PPC) in visuomotor tasks. The PPC plays a central role in visuospatial attention, multisensory integration, and motor planning. Through its bidirectional connections with premotor and somatosensory regions, it acts as a sensorimotor interface, transforming sensory inputs into goal-directed action-planning [12] and motor commands [13]. Neuroimaging evidence shows PPC activation during reaching, grasping, and pointing movements, reflecting its role in linking perception to action [14], while also contributing to later stages of motor learning by supporting visuomotor adaptation, error correction, and the updating of internal movement representations [15].

Transcranial direct current stimulation (tDCS) is a non-invasive neuromodulatory technique that modulates cortical excitability and promotes plasticity in sensorimotor networks [16]. In the context of motor learning acquisition and retention phases, most studies focus on the effects of anodal tDCS over the primary motor cortex (M1) [17]. However, in recent findings, anodal tDCS over the PPC has resulted in enhanced motor learning and faster reaction times during advanced stages of motor learning and planning [18], suggesting that PPC modulation facilitates motor refinement and consolidation. This highlights the potential for tDCS over PPC as a therapeutic intervention within multisensory frameworks aimed at optimizing motor learning outcomes.

Motor transfer refers to the influence that learning one motor skill exerts on the performance of another, typically arising when trained and untrained tasks share cognitive or motor demands [19],[20]. Rhythm-based IVR activities share visuomotor, attentional, and temporal processing demands with several standardized functional tests. Rutkowski et al. [21] showed that short-term Beat Saber training improved hand–eye coordination and reaction time using a validated transfer test battery, reinforcing the relevance of standardized motor transfer assessments to capture visuomotor improvements that extend beyond the training environment.

From this perspective, examining how multisensory and visuospatial brain regions contribute to motor learning is essential for advancing motor skill training. Herein, we combine two complementary approaches: motor training through immersive virtual reality, which provides peripersonal space exploration and multisensory inputs, and anodal stimulation of the PPC, aiming to enhance multisensory integration and visuospatial processing. We hypothesize that repetitive, rhythm-based IVR training improves performance in the game and promotes positive motor transfer to untrained tasks, assessing coordination, dexterity, and reaction time. Furthermore, we expect that the simultaneous application of tDCS during IVR training modulates these visuospatial and multisensory pathways, thereby accelerating visuomotor response speed and potentially strengthening transfer to untrained motor tasks compared to sham and IVR-only conditions. Thus, this study investigates the independent and combined effects of IVR and PPC-tDCS on visuomotor performance, response speed, and motor transfer in healthy adults, providing a targeted investigation of whether neuromodulation can enhance motor learning within complex, multisensory, and ecologically relevant virtual environments.

## 2. Materials and methods

This study was conducted in accordance with the Declaration of Helsinki, following the Consolidated Standards of Reporting Trials (CONSORT) statement, and was approved by the local research ethics committee of the Presbyterian Mackenzie University, São Paulo, Brazil (CAAE certificate: 60212822.1.0000.0084). All eligible participants received written and oral information about the study's objectives and signed an informed consent document before participating. The study was conducted at the Social and Cognitive Neuroscience Laboratory and the Physical Therapy Clinic of the Presbyterian Mackenzie University.

### 2.1. Participants

Participants were recruited to participate in the study through social media platforms like Facebook, Instagram, and WhatsApp. The present study included participants of both sexes, aged between 18 and 40 years, who were right-handed, as assessed by the Edinburgh laterality inventory [22]. Individuals with recent musculoskeletal injuries in the arms, pregnant women, skin lesions on the stimulation area, metal implants in the head, cardiac pacemakers, or recurrent seizure history were excluded from the study.

The sample size calculation was performed using the GPower (3.1.9.7) software to ensure adequate statistical power for the study. The parameters for the calculation included an effect size F = 0.5, a significance level alpha = 0.05, and a statistical power (1-β) of 0.80, with three groups and four repeated measurements per participant. Additionally, a correlation of 0.5 was assumed between the repeated measurements. The calculation indicated that a minimum of 12 participants (i.e., four per group) would be required. However, 74 participants were recruited, aiming to enhance the robustness and reliability of the results.

The recruited participants were randomly allocated to one of three groups. Group 1 (IVR): 24 participants received IVR alone; Group 2 (IVR + atDCS): 25 participants received anodal tDCS combined with IVR; Group 3 (IVR + stDCS): 25 participants received sham tDCS combined with IVR. To ensure group comparability, baseline characteristics were statistically compared using one-way ANOVA (Welch's) and Chi-square tests. Although a statistical difference was found in the continuous scores of the Edinburgh Handedness Inventory (p = 0.033), this reflects variability in the degree of lateralization rather than meaningful differences in handedness classification, as all participants were within the right-handed range (see Table 1 for participants' data according to group).

The randomization process was generated using the website www.ramdomized.org. Allocation concealment was ensured using sequentially numbered and sealed envelopes. For the groups involving tDCS treatment, participants were unaware of which stimulation they received, whether active or sham. All participants were naive to the Beat Saber game, used in the IVR training.

**Table 1.** Participant characterization data, according to the group.

Group/characteristic	IVR	IVR + atDCS	IVR + stDCS	Statistical measure
N (F/M)	24 (19/5)	25 (12/13)	25 (16/9)	χ^2^(2) = 5.14, p = 0.077
Age (mean ± SD)	20.9 ± 3.09	21.5 ± 2.60	20.8 ± 2.88	F_2,46.9_ = 0.458, p = 0.635
Edinburgh (mean ± SD)	66.7 ± 23.7	74 ±15	81.6 ± 15.5	F_2,45.5_ = 3.67, p = 0.033

Note: Data are presented as mean ± standard deviation (SD) or as absolute frequency (N). F/M = female/male participants; IVR = immersive virtual reality; atDCS = anodal transcranial direct current stimulation; stDCS = sham transcranial direct current stimulation; Edinburgh = Edinburgh Handedness Inventory score.

### 2.2. Interventions

#### 2.2.1. Transcranial direct current stimulation

The equipment delivers direct electric current and consists of four main components: electrodes (anode and cathode), an ammeter (measuring the current intensity), a potentiometer (allowing the adjustment of current intensity), and a set of three 9-volt batteries in series, resulting in a total voltage of 27 volts, to generate the applied current. This voltage is applied to the ends of a potentiometer (rheostat) with a resistance of 100 kOhms. The electrodes transmitting the electric current to the scalp consist of metal wire inside conductive rubber and wrapped in sponges soaked in saline solution.

For our stimulation sessions, the anodal electrode (5 × 5 cm) was positioned over the left parietal cortex (P3) according to the 10–20 EEG system and the cathodal electrode (5 × 7 cm) over the contralateral supraorbital area. For the active stimulation group, the device was turned on in a 30 s linear up ramp until reaching 2 mA intensity, and stimulation was delivered for 5 min before and during the Beat Saber game (~15 min) and switched off in a 30 s linear down ramp, totaling 20 min of stimulation. For the sham tDCS, the equipment was turned on in a 30 s linear up ramp until reaching 2 mA intensity; then, after 20 s of stimulation, it was turned off in a 30 s linear ramp down.

Anodal tDCS was delivered over the left posterior parietal cortex (PPC), targeting a region functionally implicated in visuomotor integration and sensorimotor transformation. The choice of a left-hemispheric montage was based on convergent evidence demonstrating hemispheric specialization of this area: neurophysiological data indicate that the left PPC exerts causal, top-down modulation over premotor regions during visuomotor processing [13], while behavioral studies show that anodal stimulation of the left, but not right, PPC selectively facilitates reaction-time improvements and late-stage motor sequence learning [18]. Given the demands of the training task, which require rapid visuomotor mapping and dynamic spatial updating, the left PPC is the most appropriate target.

#### 2.2.2. Immersive virtual reality

For IVR, the equipment used was the PlayStation 4 (PS4), a video game developed by Sony in 2013, with motion sensor technology embedded in the controller and capable of rendering 4K images. Two controllers with manual sensors are used as lightsabers to cut colored blocks that appear on the screen in rhythm with the music corresponding to the game level. The game has three difficulty levels (easy, medium, and hard). In this study, level 1 of the game was used for the first three days to facilitate the acquisition and consolidation of task learning. Level 2 was used to enhance motor learning on the fourth and fifth days.

The game's score is based on the accuracy and angle of the cuts made on blocks in the virtual environment. The scoring system is subdivided into three main components: 70 points are awarded based on the initial cut angle, which must be greater than 100° to achieve the maximum score; 30 points are given for the follow-through, which must maintain an angle greater than 60° after the cut; and up to 15 points are earned based on precision when cutting near the center of the block. Additionally, the player's total score is amplified by a combo multiplier, which increases with consecutive successful cuts. A poorly executed cut, outside the direction indicated by the block, or a failure to cut, results in point reduction and a break in the combo multiplier. If the player fails to hit any block, either due to timing or directional errors, they receive a miss, resulting in 0 points and an immediate combo break. Accumulating failures can result in a final score of zero.

### 2.3. Motor evaluation tests

*Box and Block test:* This test evaluates manual dexterity [23]. A wooden box with a divider, containing 150 colored blocks, was placed on a table. The participant was instructed to transfer the blocks from one side of the box to the other as quickly as possible. Both the right and left hands were tested individually. The time taken to complete the task was recorded using a stopwatch, and the score was calculated as the number of blocks transferred per minute.

*Plate Tapping test:* This test assesses speed and coordination [21]. Two circles and a rectangle are placed on a table. The two discs are 60 cm apart, with the rectangle placed equidistant from them. The non-dominant hand rests on the rectangle, while the participant uses the dominant hand to move back and forth between the discs as quickly as possible. The time, measured in seconds, is recorded with a stopwatch until the participant completes 25 cycles.

*Ruler Drop test:* This test assesses reaction time [24]. The participant sits on a chair with their fingers positioned at the zero mark on a ruler. The assessor releases the ruler, and the participant must close their fingers around it as quickly as possible. The reaction time is determined by the point on the ruler where the participant's fingers grasp it. In our protocol, participants performed the task with both right and left hands.

*Trail Making test (Part A):* This test evaluates visual coordination and attention [25]. The participant is instructed to connect a sequence of randomly scattered numbers on a sheet of paper in ascending order, completing the task as quickly and accurately as possible. The time taken to finish the task is measured in seconds using a stopwatch. In our protocol, participants performed this task with the dominant (right) hand.

### 2.4. Procedure

The study followed a three-arm, randomized, sham-controlled, single-blind design. All participants received verbal and written information and signed the informed consent before starting the procedure. Participants attended the research laboratory for one session per day over five consecutive days. In the first session, participant assessments were conducted, including the collection of demographic data (such as age, gender, and education level). Further, at both first and last sessions, i.e., days 1 and 5, motor evaluation tests were administered before and after intervention, namely, the Plate Tapping test, Ruler Drop test, Trail-Making test A, and Box and Block test (see Figure 1).

Regarding the intervention, for the groups who received tDCS treatment, the electrodes were initially placed over the scalp, and the stimulation (either anodal or sham) was initiated 5 min before and during the entire IVR session. After the session ended, the tDCS was turned off, and the participant verbally answered the questionnaire on tDCS side effects [26]. Furthermore, to evaluate blinding effectiveness, participants had to indicate which type of tDCS they believed they had received (active or sham) on a scale from 0 (no certainty) to 5 (maximum certainty). These responses were then compared with the actual stimulation condition administered. The IVR group participants skipped those tDCS steps; the IVR headset was immediately fitted, and they had no stimulation questionnaires to answer at the end.

For the IVR session, the participant was then positioned in the center of the room, standing and facing a screen and the PS4 sensor, while holding two controllers that tracked arm movements. The participant played the game Beat Saber for 15 min. Fatigue was assessed using the Borg scale before and after each IVR session. In total, each IVR group session lasted for 15 min, whereas the other group sessions lasted for approximately 25 min.

**Figure 1.**
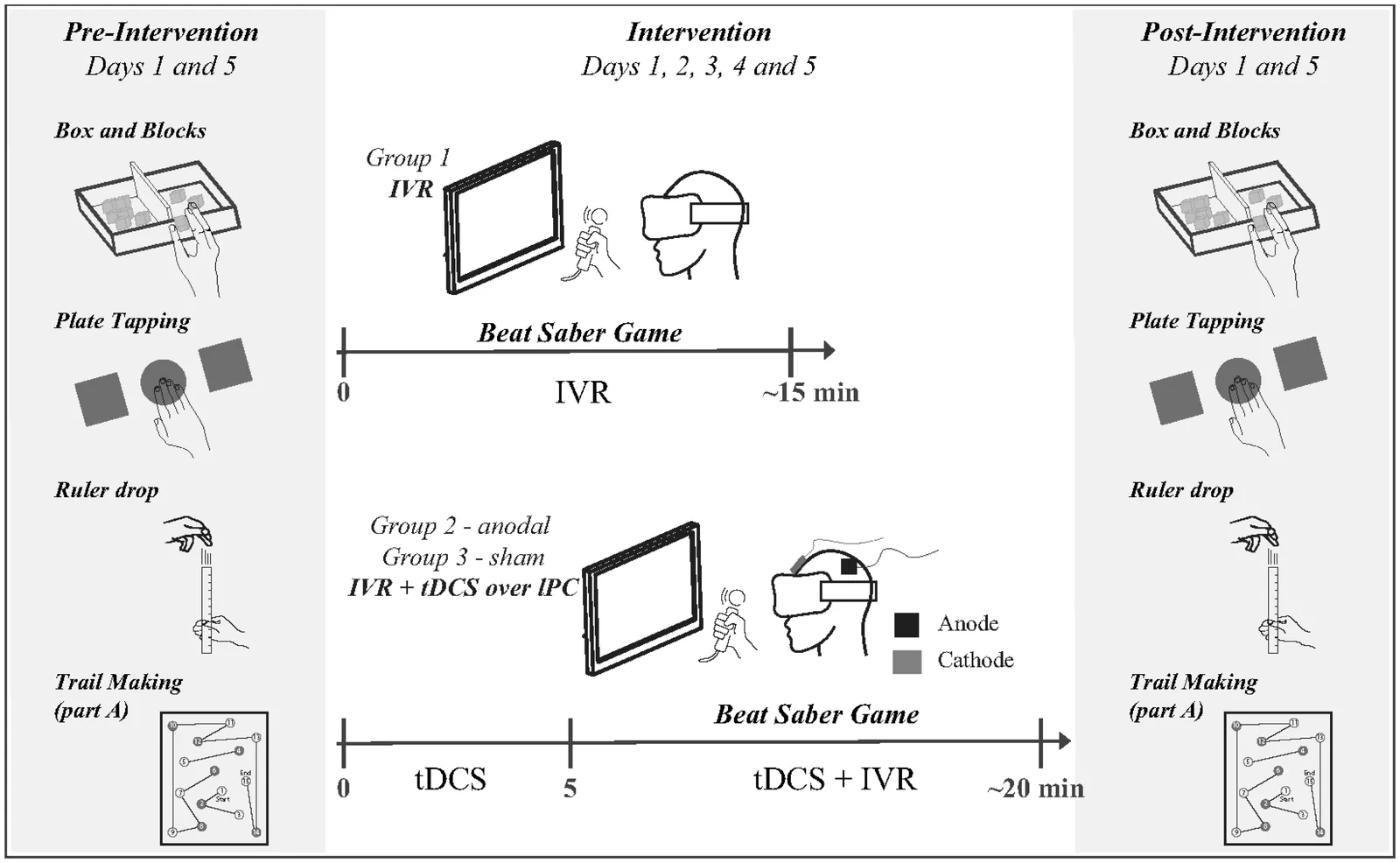
Study procedure: Participants underwent 5 days of intervention consisting of a motor game, the Beat Saber game, performed in an immersive virtual reality (IVR) environment alone (Group 1) or combined with anodal (Group 2) or sham tDCS (Group 3) over the left parietal cortex. At days 1 and 5, they performed four motor tasks pre- and post-intervention: Box and Blocks, Plate Tapping, Ruler Drop, and part A of the Trail Making task.

### 2.5. Statistical analysis

All data followed a normal distribution, and sphericity was not violated. We analyzed the measures from each test performed before and after the training intervention using a mixed-design analysis of variance (mixed-design ANOVA). The dependent and independent variables for each test are described below. For all tests, we assumed an alpha level of 5%, and when appropriate, Bonferroni-corrected post hoc analyses were performed.

For the Box and Block test, we analyzed the total number of blocks moved during 60 s, considering Time (D1_pre vs. D1_post vs. D5_pre vs. D5_post) and Hand (left vs. right) as within-subjects factors, and Group (control vs. atDCS vs. stDCS) as the between-subjects factor.

For the Plate Tapping test, we used the best time to complete the task (in seconds) as the dependent measure and Time (D1_pre vs. D1_post vs. D5_pre vs. D5_post) and Group (control vs. atDCS vs. stDCS) as the within- and between-subjects factors, respectively.

For the Ruler drop test, we analyzed the distance (in centimeters) that the ruler traveled before the participant successfully caught it. We considered Time (D1_pre vs. D1_post vs. D5_pre vs. D5_post) and Hand (left vs. right) as within-subjects factors, and Group (control vs. atDCS vs. stDCS) as the between-subjects factor.

For the Trail Making test, the time to complete the task (in seconds) was used as the dependent variable and Time (D1_pre vs. D1_post vs. D5_pre vs. D5_post) and Group (control vs. atDCS vs. stDCS) as within- and between-subjects factors, respectively.

Finally, we also analyzed the punctuation in the Beat Saber game and fatigue. For the game score, we considered Day (D1 vs. D2 vs. D3 vs. D4 vs. D5) and Group (control, vs. atDCS vs. stDCS) as the within- and between-subjects factors, respectively. For the fatigue, we used the Borg Scale scores as the dependent measure and considered Day (D1 vs. D2 vs. D3 vs. D4 vs. D5) and Moment (pre- vs. post-intervention) as within subjects factor and Group (control vs. atDCS vs. stDCS) as the between-subjects factor.

## 3. Results

### 3.1. Motor transfer tests

#### 3.1.1. Box and Block test

For the Box and Block test, we found significant main effects for Time and Hand (please see Table 2). Bonferroni-corrected post hoc analysis of Time showed that participants exhibited improved performance following the interventions. This improvement was already significant on D1_post (p < 0.001) and was sustained up to D5, as evidenced by significant differences between D1_pre and D5_pre (p < 0.001), but not between D1_post and D5_pre (p = 1). Additionally, the final training session further improved performance (D5_pre ≠ D5_post, p < 0.001). There was also an incremental improvement when comparing D1_pre with D5_post (p < 0.001) (see Figure 2). Regarding the laterality effect, participants performed better with their right hand (72.5 ± 0.996) compared to their left hand (70.2 ± 0.944).

No main effect was found for Group, nor for any interaction.

#### 3.1.2. Plate Tapping test

For the Plate Tapping test, we found a significant main effect for Time. Bonferroni-corrected post hoc analysis of Time revealed continuous improvement from day 1 to day 5. As depicted in Figure 2, participants became faster over time.

No main effect was found for Group, nor for the interaction Group*Time. See Table 2 for detailed statistical results.

#### 3.1.3. Ruler drop test

For the Ruler drop test, we found significant main effects for Time and Hand. Bonferroni-corrected post hoc analysis of Time revealed continuous improvement from day 1 to day 5 (D1_pre ≠ D1_post (p = 0.020) and ≠ D5_pre (p < 0.001); D1_post ≠ D5_pre (p = 0.003). This improvement plateaued after a few days of training (D5_pre = D5_post (p = 1)); however, performance remained significantly better when comparing D1_post with D5_post (p = 0.006). Regarding the Hand effect, curiously, participants overall showed a better performance when using their left hand (9.53 ± 0.470) compared to the right hand (10.57 ± 0.408) (see Figure 2).

No main effect was found for Group, nor for the interactions (see Table 2 for detailed statistics).

#### 3.1.4. Trail Making

For the Trail Making test, there was a significant main effect for Time. Bonferroni-corrected post hoc analysis showed that participants performed the task faster following the interventions. This improvement was already significant on D1_post (p < 0.001) and was sustained up to D5, as evidenced by significant differences between D1_pre and D5_pre (p < 0.001), but not between D1_post and D5_pre (p > 0.275). Additionally, the final training session further improved performance (D5_pre ≠ D5_post, p < 0.001). There was also an incremental improvement when comparing D1_pre with D5_post (p < 0.001) (see Figure 2).

No main effect was found for Group, nor for the interaction Group*Time (see Table 2 for detailed statistics).

**Figure 2.**
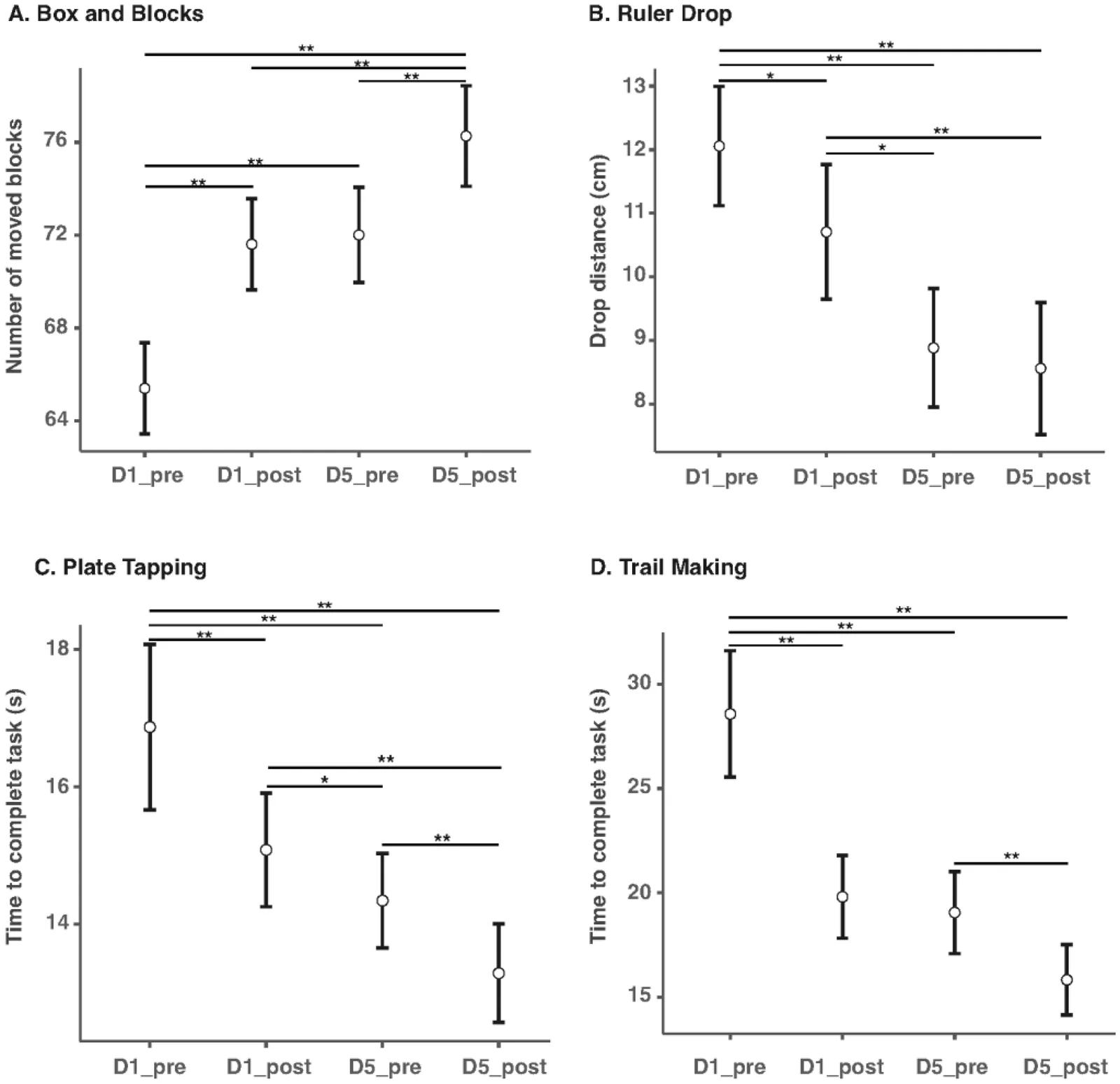
Transfer tasks results. A) Box and Blocks plots the mean number of blocks moved in 60 s according to the different time points. B) Ruler grip plots the mean distance the ruler dropped, measured in centimeters, before the participant catches it, in the different time points of evaluation. C) Plate tapping plots the mean time (in seconds) to conclude the task in each time point. D) Trail making plots the mean time (in seconds) to conclude the task in each time point. For all plots in all panels, bars represent 95% CI; (*) p < 0.05; (**) p ≤ 0.001.

**Table 2.** Performance and mixed-design ANOVA outcomes for the motor transfer tasks across groups and time points.

**Groups**	**IVR (mean ± SE)**	**atDCS (mean ± SE)**	**stDCS (mean ± SE)**	**Mixed-design ANOVA effects**
**Box and Block (blocks/60 s)**	
D1_pre	66.1 ± 1.73	64.3 ± 1.69	65.8 ± 1.69	Time: F_3,213_ = 97.917, p < 0.001, η_p_^2^ = 0.580
D1_post	71.4 ± 1.73	71.4 ± 1.69	71.9 ± 1.69	Hand: F_1,71_ = 25.504, p < 0.001, η_p_^2^ = 0.264
D5_pre	70.8 ± 1.81	72.1 ± 1.77	73.1 ± 1.77	Group: F_2,71_ = 0.106, p = 0.899, η_p_^2^ = 0.003
D5_post	75.6 ± 1.91	76.3 ± 1.87	76.9 ± 1.87	Interactions: • Group × Time: F = 0.870, p = 0.518 • Group × Hand: F = 0.593, p = 0.556 • Time × Hand: F = 0.459, p = 0.711 • Time × Group × Hand: F = 0.804, p = 0.568
**Plate Tapping (s)**				
D1_pre	17.9 ± 1.06	16.8 ± 1.04	16 ± 1.04	Time: F_3,213_ = 29.941, p < 0.001, η_p_^2^ = 0.297
D1_post	15.6 ± 0.73	14.7 ± 0.71	14.9 ± 0.71	Group: F_2,71_ = 1.01, p = 0.370, η_p_^2^ = 0.028
D5_pre	15.2 ± 0.61	13.6 ± 0.59	14.3 ± 0.59	Group × Time: F_6,213_ = 0.612, p = 0.721, η_p_^2^ = 0.017
D5_post	13.9 ± 0.63	12.8 ± 0.62	13.1 ± 0.62	
**Ruler drop (cm)**				
D1_pre	(L) 11.38 ± 1.07 (R) 13.21 ± 0.97	(L) 11.94 ± 1.05 (R) 10.92 ± 0.95	(L) 12.62 ± 1.05 (R) 12.28 ± 0.95	Time: F_3,213_ = 21.694, p < 0.001, η_p_^2^ = 0.234
D1_post	(L) 9.15 ± 1.12 (R) 12.23 ± 1.01	(L) 10 ± 1.10 (R) 11.26 ± 0.99	(L) 10.48 ± 1.10 (R) 11.12 ± 0.99	Hand: F_1,71_ = 7.079, p = 0.010, η_p_^2^ = 0.091
D5_pre	(L) 8.13 ± 1.06 (R) 8.65 ± 0.92	(L) 7.36 ± 1.04 (R) 10 ± 0.89	(L) 9.86 ± 1.04 (R) 9.30 ± 0.89	Group: F_2,71_ = 0.171, p = 0.843, η_p_^2^ = 0.005
D5_post	(L) 8.19 ± 1.27 (R) 8.52 ± 1.05	(L) 8.14 ± 1.25 (R) 9.18 ± 1.03	(L) 7.18 ± 1.25 (R) 10.14 ± 1.03	Interactions: • Group × Time: F = 0.368, p = 0.898 • Group × Hand: F = 0.328, p = 0.722 • Time × Hand: F = 1.050, p = 0.371 • Time × Group × Hand: F = 1.993, p = 0.068
**Trail Making (s)**				
D1_pre	31.3 ± 2.67	26.1 ± 2.62	28.2 ± 2.62	Time: F_3,213_ = 129.527, p < 0.001, η_p_^2^ = 0.646
D1_post	21.6 ± 1.75	18.1 ± 1.71	19.7 ± 1.71	Group: F_2,71_ = 0.857, p = 0.429, η_p_^2^ = 0.024
D5_pre	20.7 ± 1.74	18.1 ± 1.70	18.4 ± 1.70	Group × Time: F_6,213_ = 0.823, p = 0.553, η_p_^2^ = 0.023
D5_post	16.9 ± 1.49	15.1 ± 1.46	15.5 ± 1.46	

Note: Data for each task score are presented as mean ± standard error (SE). IVR: immersive virtual reality; atDCS: anodal transcranial direct current stimulation; stDCS: sham transcranial direct current stimulation; D1: day 1; D5: day 5; pre: pre-intervention; post: post-intervention assessment; s: seconds; cm: centimeters. (L) and (R) in the Ruler drop test denote left- and right-hand performances, respectively.

### 3.2. Beat Saber performance

Concerning the score at the Beat Saber game, we found a significant effect for Day (F_4,284_ = 165.88, p < 0.001, η_p_^2^ = 0.700). Bonferroni-corrected post hoc analysis showed a gradual increase of performance according to time, with all paired comparisons having a p < 0.001, except when comparing days 2 and 3 (p = 0.046) and days 4 and 5 (p = 0.074) (see Figure 3). Means and SE are depicted in Table 3.

**Figure 3.**
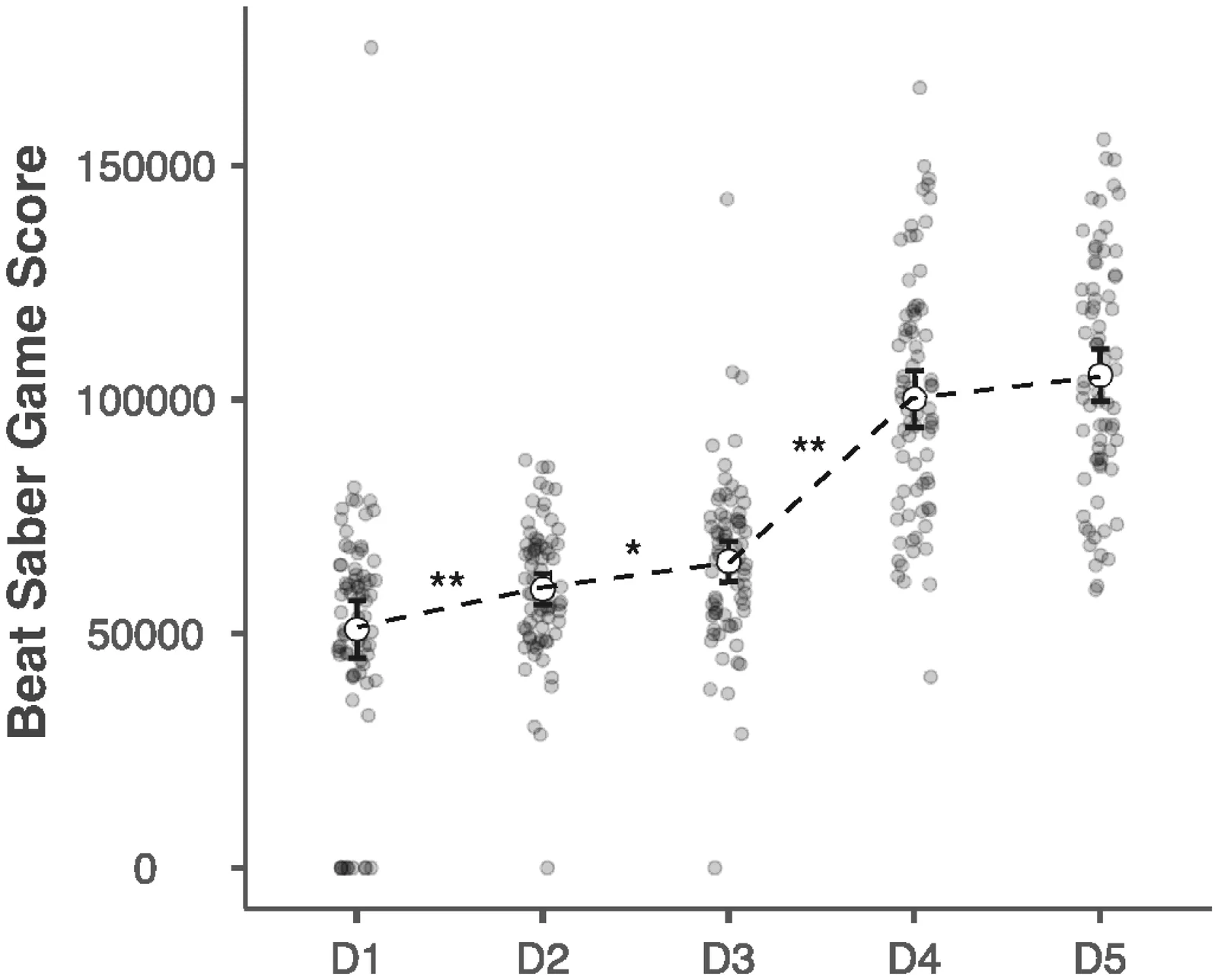
Beat Saber game score of all participants and group average for each training day. The dashed line shows the gradual increase in performance according to time, with a plateau from days 4 to 5. Bars represent 95% CI; (*) p < 0.05; (**) p ≤ 0.001.

### 3.3. Fatigue assessment

As for the fatigue assessment, we found a significant effect of Moment (F_1,71_ = 36.052, p < 0.001, η_p_^2^ = 0.337), meaning that participants felt more fatigue after the intervention (2.88 ± 0.218) compared to before (1.97 ± 0.204). No main effects were found for Day and Group nor for any interactions. Means and SE considering day and moment are depicted in Table 3.

**Table 3.** Mean scores on the Beat Saber game in each IVR session/day and respective fatigue score assessed with Borg Scale.

**Days**		**D1**	**D2**	**D3**	**D4**	**D5**
**Points on the *Beat Saber* game**		50 964 (± 3110)	59 507 (± 1675)	65 452 (± 2166)	100 156 (± 3024)	105 191 (± 2827)
**Fatigue (Borg scale)**	**Pre**	1.68 (± 0.256)	2.22 (± 0.281)	1.98 (± 0.253)	1.75 (± 0.245)	2.21 (± 0.283)
	**Post**	2.86 (± 0.256)	2.86 (± 0.275)	2.70 (± 0.255)	2.89 (± 0.276)	3.11 (± 0.285)

Note: Values represent mean (± SE). Data for each task score are presented as mean ± standard error (SE). D1: day 1; D2: day 2; D3: day 3; D4: day 4; D5: day 5; pre: pre-intervention; post: post-intervention assessment.

### 3.4. TDCS blinding and safety

Finally, considering safety and blinding of tDCS, the safety analyses indicated that tDCS was well-tolerated, with no significant differences between groups for headache (χ² = 0.00, p = 0.957), neck pain (χ² = 4.00, p = 0.135), or scalp pain (χ² = 1.34, p = 0.513). Similarly, reports of hearing impairment (p = 0.141), concentration difficulties (p = 0.238), and acute mood changes (p = 0.310) were equivalent across conditions, while no cognitive impairment occurred. Although scalp burning frequency did not reach statistical significance (χ² = 5.36, p = 0.147), a borderline trend was observed (τb = −0.265, p = 0.061), with higher mild reports in the active group (24% vs. 4.2%). Overall, adverse events were predominantly mild, with no serious adverse events reported.

Blinding integrity was rigorously maintained throughout the intervention. Participants were unable to correctly identify their treatment allocation above chance levels on both day 1 (χ² = 2.46, p = 0.117) and day 5 (χ² = 1.12, p = 0.291). Furthermore, the level of certainty in their guesses did not differ significantly between the Anodal and Sham groups at the beginning of the study (t(47) = −0.108, p = 0.915 (Anodal: 3.20 ± 1.61; Sham: 3.25 ± 1.65)), nor at the end of the 5-day protocol (t(47) = 1.07, p = 0.288 (Anodal: 3.96 ± 1.17; Sham: 3.58 ± 1.28)). These findings, confirmed by nonparametric analyses, demonstrate that the sham procedure effectively masked the stimulation condition, ensuring the internal validity of the clinical outcomes.

## 4. Discussion

Our experiment examined the effects of motor training by means of playing an IVR game over five consecutive days, both independently and combined with active or sham tDCS over the left parietal cortex, on task performance and motor transfer. Motor performance was assessed daily through scores in the game played in the IVR, the Beat Saber game. In turn, motor transfer was evaluated using a battery of tests, specifically, the Box and Block, Ruler Drop, Plate Tapping, and Trail Making tests, which were administered before and after the first and fifth intervention sessions. Importantly, these findings underscore the role of immersive virtual reality training in promoting motor learning within complex, multisensory, and ecologically relevant environments, while also providing additional insights into how neuromodulation applied over the posterior parietal cortex may interact with multisensory and visuospatial processes during motor learning.

As expected, training yielded significant effects on game scores, showing that IVR practice led to continuous improvement in performance irrespective of stimulation conditions. Additionally, we found significant motor transfer effects in all tests of the applied battery, suggesting that the benefits of training extended beyond the specific context of the Beat Saber game, positively impacting generalizable motor skills.

The observed improvement in game scores over time is not surprising, as it is aligned with well-established principles of motor learning, whereby repetitive practice effectively facilitates motor skill acquisition [27],[28]. This process involves synaptic reorganization and neuroplasticity [29], which contribute to the formation of durable motor memories [30]. Repetitive, rhythm-based actions in Beat Saber likely refined motor performance and led to greater task proficiency. Importantly, the performance improved progressively up to the fourth training day, followed by stabilization between days four and five, suggesting a learning plateau.

Most interestingly, the training promoted improvements in other motor tasks beyond the trained game, demonstrating a positive motor transfer effect. Specifically, participants became faster in the Box and Block, Plate Tapping, Trail Making, and Ruler Drop tests, all of which assess different components of motor control, such as upper-limb motor coordination, dexterity, reaction time, and cognitive–motor integration [23],[24],[31]. These findings suggest that VR training, even when using a commercial game that does not directly mimic the post-training tasks, can lead to meaningful transfer of motor skills.

Motor transfer effects are often discussed within the frameworks of the Identical Elements and the Transfer-Appropriate Processing theories [19],[20]. In our experiment, although Beat Saber did not directly mirror the assessed tasks, its rhythm-based movements and decision-making demands share fundamental similarities with the motor and cognitive requirements of the transfer tasks, facilitating skill generalization and effective learning.

Regarding the Box and Blocks test, which focuses on manual dexterity and coordination, the number of blocks moved increased significantly after the first IVR session, suggesting a prompt enhancement that was sustained throughout the consecutive training sessions, as observed in the pre-session of day 5. Still, performance continued to increase after the final training day, as evidenced by significantly higher scores in D5_post compared to D5_pre. These observed transfer effects are consistent with the rapid hand movements and timing required during Beat Saber gameplay. A similar pattern of improvement was observed in the Trail Making test, which assesses cognitive–motor integration: participants performed better after the first training session, with sustained improvements up to day 5 and further enhancement after the final session. The dynamic, fast-paced nature of the game likely enhanced attention, task-switching, and cognitive flexibility, which are essential for this task [31].

In the Plate Tapping test, which assesses bilateral coordination and reaction speed, participants showed continuous improvement across assessment sessions, indicating motor learning facilitation throughout the IVR training. These transfer effects align with the alternating movements required in Beat Saber, where players must quickly respond to visual and spatial cues with both hands, thus likely honing participants' ability to perform alternating actions with precision, enhancing bilateral coordination and speed. Similarly, participants demonstrated continuous improvement in the Ruler Drop Test, with faster reaction times as training progressed. However, performance plateaued after the D5_pre session, suggesting that participants had reached their peak improvement by this point. Interestingly, this corresponds to the improvements in game performance, which also showed no incremental enhancement from day 4 to day 5.

In sum, our positive motor transfer from the IVR game suggests that repetitive, rhythm-based, and timed actions, with emphasis on hand–eye coordination, likely contributed to enhanced manual dexterity, motor control, hand coordination, and stimulated cognitive processing that translated to other real-world tasks, leading to improved performance on the time-sensitive cognitive-motor tasks employed in our test battery. Altogether, these results suggest both immediate and cumulative long-term effects.

These findings are consistent with theories emphasizing the role of overlapping motor actions and cognitive processes in facilitating skill transfer [19],[32]. These overlaps likely facilitated the transfer of skills such as manual dexterity, reaction speed, and cognitive flexibility, supporting transfer to other real-world tasks.

In our case, the cognitive demands of *Beat Saber*, including attention, reaction time, and spatial awareness, mirror the cognitive processes required for tasks like the Trail Making Test and the Ruler Drop Test. This suggests that the game's combination of motor and cognitive challenges created an ideal learning environment for transferring skills to tasks that required quick reactions, coordination, and cognitive-motor integration.

The transfer of motor skills from VR training to real-world settings using commercial games has been tested in different sports, including table tennis [33], dart throwing [34], and golf putting [35]. These experiments report mixed results. While VR training improved table tennis performance compared to no practice [33], dart throwing performance decreased following VR practice but improved with real-world practice [34]. Finally, Markwell et al. [35] found that VR and real-world golf putting practice resulted in similar golf swing kinematics and improved accuracy at similar rates. Our results extend previous findings by demonstrating that meaningful transfer can occur even with a commercial IVR game when cognitive and motor elements align with real-world tasks, without requiring direct correspondence.

Moreover, we also analyzed laterality effects for the Box and Block and the Ruler Drop tests, and both yielded significant main effects independent of time. In the Box and Block Test, participants performed better with their right (dominant) hand compared to their left hand, consistent with the well-documented advantage of the dominant hand in tasks requiring precision and dexterity [36],[37]. This dominance effect is likely due to greater neural specialization and motor skill refinement in the hemisphere contralateral to the dominant hand [38].

Interestingly, in the Ruler Drop Test, participants performed better with their left (non-dominant) hand compared to their right hand. This contrasts with typical dominance effects observed in motor tasks, including the Ruler Drop test [39]. While unexpected, this pattern may reflect the training of bilateral and rhythm-based movements within the game, which could have had a stronger effect on enhancing the non-dominant hand's reaction time and motor control, potentially promoting interhemispheric communication [40],[41]. Previous studies show that the non-dominant hand may benefit more from practice, as it has greater room for improvement compared to the already-skilled dominant hand [42]. However, the absence of a significant interaction between laterality and time limits this interpretation, and further research is needed to determine whether this laterality effect is specific to the task type and how exactly the demands of IVR training influence the skills required to perform it.

We hypothesized that anodal tDCS over the left PPC could boost Beat Saber performance and enhance motor transfer, given its role in motor coordination and visuospatial processing [43]. Compared to M1 or PMC, the effects of PPC stimulation are underexplored, prompting investigation of its potential to support visuospatial attention during training. However, no significant differences were observed between active, sham, and no-stimulation groups, and several factors compete to explain this lack of measurable effects, including the stimulation site, possible ceiling effects, and several stimulation parameters.

Regarding the stimulation site, although PPC stimulation may influence attentional processes, it might not directly modulate motor execution or learning to the same extent as areas like M1 or PMC. Previous research showed that anodal stimulation over the PPC increased performance in a serial reaction task only when applied during the late stage of learning [18]. Moreover, recent reviews indicate that targeting M1 has the greatest potential for enhancing learning and retention in motor training, followed by stimulation of the left premotor cortex and the cerebellum, whereas stimulating the parietal areas has been less explored and shows mixed results [44]–[46]. We also found no effect on fatigue, contrary to previous studies on motor tasks and muscular endurance following stimulation, though these effects are mostly observed after stimulation over M1 [45].

Regarding other stimulation parameters, current intensity, duration, and electrode placement may also have contributed to the lack of observable effects. The determination of optimal parameters for tDCS is often debated due to many reports on the non-replicability of findings, which is also partially due to individual variability [47],[48]. Additionally, some evidence suggests that the effects of tDCS are more pronounced when applied during specific phases of learning or consolidation [18],[41]. Therefore, applying stimulation either immediately before or after training, rather than during gameplay, could potentially yield different outcomes.

Still, beyond our specific experimental design, some studies have also questioned the robustness of tDCS effects in general. Recent large-scale trials and meta-analyses have failed to consistently demonstrate reliable behavioral improvements across cognitive and motor domains, raising concerns about its general efficacy and reproducibility [49],[50].

Finally, ceiling effects cannot be ruled out. Significant improvements were found across all groups, suggesting that the IVR training alone was highly effective in promoting motor learning and transfer. Although the constant improvement in the game performance up to day 4 argues against this possibility, several studies show that combining neuromodulation either to drugs or tasks that increase motor area activation and performance often results in null or opposite results, likely due to homeostatic effects [51],[52]. Moreover, considering the motor transfer assessments, the rapid skill acquisition and performance plateau observed in the Ruler Drop and Plate Tapping suggest that limited room remained for further improvement via neuromodulation.

Therefore, although the PPC may support motor acquisition, retention, and transfer, our experiment provided no evidence to support it. As the available literature on the topic remains sparse, the specific advantages of modulating this area for improving motor learning and transfer remain unclear.

## 5. Conclusions, limitations, and future directions

Our study demonstrates that IVR training using a commercially available device can promote motor learning and positive transfer to untrained tasks. These results highlight the potential of IVR as a versatile and accessible tool for motor training and skill development, particularly in contexts where traditional methods may be impractical or unavailable. However, the lack of significant differences between stimulation conditions suggests that anodal tDCS over the left PPC may not provide additional benefits in this context, or that methodological factors limited its efficacy.

One limitation of our study is the use of a single polarity (anodal) and target area (left PPC). We did not compare stimulation over the right hemisphere or explore other target areas, limiting our ability to determine whether the lack of effects was due to a ceiling effect, suboptimal target selection, or stimulation parameters such as intensity, duration, and montage. Future studies should explore different parameters, including cathodal stimulation, to clarify whether ceiling effects influenced outcomes.

Regarding our evaluation timeline, we assessed motor performance immediately before and after training sessions on days 1 and 5, but did not evaluate long-term assessments beyond the training period. While the pre-training assessment on day 5 provides an indication of retention, it does not fully align with standard retention testing protocols, which recommend assessments at least 24 hours after the final training session to evaluate long-term skill retention [53],[54]. Future research should incorporate follow-up assessments beyond the training period, including evaluations at longer intervals (e.g., days, weeks, or months), to evaluate the longevity and consolidation of motor improvements in VR-based motor training contexts.

Beyond the assessment schedule, the study design is also constrained by the absence of a completely untreated (no-intervention) control condition, making it challenging to disentangle the specific contribution of immersive virtual reality practice via Beat Saber from the general effects of repeated exposure to the motor assessment battery, such as task familiarization and strategy optimization, or placebo effects. Consequently, although the robust time effects strongly suggest that the protocol as a whole promoted motor learning and transfer, the data cannot conclusively attribute these improvements solely to the IVR training, and the relative weight of IVR versus task‑specific practice remains unclear. In addition, other potential confounding factors such as motivation, expectancy effects, or differential engagement cannot be entirely excluded. Future studies should incorporate a baseline control group to definitively isolate the independent effects of IVR-based training.

An additional point of consideration is the absence of sex-stratified randomization. Although current evidence does not consistently support sex as a major determinant of motor learning outcomes [55],[56], sex-specific effects cannot be entirely excluded. Nonetheless, our baseline analysis confirmed that sex distribution did not significantly differ among the three groups, minimizing the potential confounding impact on our findings.

Finally, while our findings demonstrate the potential of IVR for motor training, further research is needed to explore its applicability across different populations, such as individuals with motor impairments or athletes, and to identify task and environmental factors that optimize transfer.

In sum, while no significant differences were observed between our active, sham, and no-tDCS groups, our positive motor transfer results highlight the potential of IVR training alone to enhance motor performance and facilitate motor transfer. Future research should investigate the long-term effects of IVR-based motor training, explore stimulation targets beyond the left PPC, and refine stimulation parameters to optimize outcomes. Moreover, expanding VR applications to clinical and rehabilitation settings could provide further insight into its effectiveness across diverse populations. While VR is unlikely to replace traditional training entirely, its ability to enhance engagement and support skill transfer makes it a valuable tool in motor learning research and practice.

## Use of AI tools declaration

The authors declare they have not used Artificial Intelligence (AI) tools in the creation of this article.
